# Mutations differentially affecting the coronavirus Mac1 ADP-ribose binding and hydrolysis activities indicate that it promotes multiple stages of the viral replication cycle

**DOI:** 10.1128/jvi.00623-25

**Published:** 2025-07-30

**Authors:** Joseph J. O'Connor, Anuradha Roy, Reem Khattabi, Catherine Kerr, Nancy Schwarting, Yousef M. Alhammad, Philip Gao, Xiaoming Zhang, Xufang Deng, Anthony R. Fehr

**Affiliations:** 1 Department of Molecular Biosciences, University of Kansas 4202, Lawrence, Kansas, USA; 2 Infectious Disease Assay Development (IDAD) Core, University of Kansas 4202, Lawrence, Kansas, USA; 3 Molecular Structures Group, University of Kansas 4202, Lawrence, Kansas, USA; 4 Department of Physiological Sciences, College of Veterinary Medicine, Oklahoma State University 7618, Stillwater, Oklahoma, USA; 5 Oklahoma Center for Respiratory and Infectious Diseases, Oklahoma State University 7618, Stillwater, Oklahoma, USA; University of Kentucky College of Medicine, Lexington, Kentucky, USA

**Keywords:** coronavirus, SARS-CoV-2, macrodomain, Mac1, ADP-ribose, non-structural protein 3

## Abstract

All coronaviruses (CoVs) encode a conserved macrodomain, termed Mac1, in non-structural protein 3 (nsp3) that binds and hydrolyzes ADP-ribose covalently attached to proteins. Mac1 is a key virulence factor that counters antiviral ADP-ribosyltransferase (PARP) activity. Previously, we found that MHV strain JHM (JHMV) with a mutation in the adenine binding site, JHMV-D1329A, was extremely attenuated in all tested cell types as opposed to JHMV-N1347A, which only has a replication defect in bone marrow–derived macrophages (BMDMs). Interestingly, an N1347A/D1329A double mutant was unrecoverable, indicating an essential role for Mac1 in JHMV infection. We hypothesized that these mutations may impact different stages of the MHV life cycle. First, to clarify how these mutations affected the biochemical activities of Mac1, we generated Mac1 proteins encoding the same mutations. As expected, the D-A mutation was extremely defective in ADP-ribose binding but maintained enzyme activity. In contrast, we previously found that the N-A mutation had WT levels of ADP-ribose binding but low enzyme activity, confirming that these mutations differentially affect the biochemical functions of Mac1. Following infection, D1329A displayed a large defect in the accumulation of viral RNA compared to WT or N1347A in all cells tested. Alternatively, N1347A infection produced normal levels of viral RNA but produced reduced levels of viral protein in interferon-competent bone marrow–derived macrophages (BMDMs). These results suggest that Mac1 ADP-ribose binding and enzymatic activities promote different stages of the viral life cycle, demonstrating the critical importance of Mac1 for JHMV replication.

**IMPORTANCE:**

Over the last three decades, coronaviruses have repeatedly demonstrated their potential to become significant veterinary and public health threats. Zoonotic transmission of the myriad known coronavirus strains will remain a concern, regardless of the advances in vaccines and treatment. One difficulty in anticipating the next coronavirus outbreak is its diverse lineage and high propensity for mutation and recombination. The coronavirus macrodomain, Mac1, is conserved among all known coronaviruses and is also conserved in the *Togaviridae* and *Hepeviridae* families. Mac1 is a key factor in viral replication and pathogenesis, but its role in the replication cycle remains unclear. A deeper investigation of Mac1 function will identify conserved antiviral mechanisms and aid in the development of Mac1 inhibitors that represent a novel strategy for antiviral therapeutics.

## INTRODUCTION

Coronaviruses (CoVs) are large, positive-sense ssRNA viruses with unusually large genomes ranging from 28 to 33 kb. Subdivided into four genera (alpha, beta, gamma, and delta), coronaviruses cause disease of clinical and veterinary significance in mammals and birds. Deadly infections in piglets with porcine endemic diarrhea virus illustrate the significant economic burden coronaviruses impose on the livestock industry (1); however, recent history demonstrates zoonotic transmission of CoVs to be an even greater economic and public health threat. Between 10 and 30% of circulating common cold viruses in humans are caused by CoVs like hCoV-229E and hCoV-OC43, which have a likely origin in zoonotic transmission from livestock (2, 3). The emergence of three highly pathogenic human CoVs within as many decades has placed the threat of novel zoonotic CoVs near the forefront of pandemic concern (4, 5). Efforts to determine the functions of highly conserved CoV proteins will aid in developing novel therapies to help mitigate future outbreaks of divergent CoVs.

The CoV genome is organized into two major segments encoding the non-structural and the structural proteins. Structural proteins, such as the spike and nucleocapsid protein, are encoded in transcriptionally “nested” ORFs at the 3’ end of the genome. The non-structural proteins (nsps) are encoded at the 5’ end in two large co-terminal ORFs that comprise about two-thirds of the genome. Nsps are translated as large polyproteins from either ORF1a or from ORF1ab, which are then proteolytically processed into each of the functional nsps. Translational regulation between these two ORFs is achieved through ribosomal frameshifting bypassing a conserved stop codon. It is thought that this strategy is meant to maintain optimal stoichiometric ratios between proteins important for blocking immunity or establishing viral infection and the proteins that constitute the RNA replication machinery (6).

Nsp3 is the largest of the coronavirus non-structural proteins with a mass of approximately 220 kDa. Nsp3 contains numerous domains with important characterized functions. These include polyprotein cleavage and deubiquitinase (DUB) activity of PL^pro^, ssRNA and nucleocapsid binding of Ubl1, ADP-ribosylhydrolase activity of the conserved macrodomain, and formation of replication organelles (ROs) (7–10). Most notably, nsp3–4 dimers form large hexamers that, on one side of the ER double membrane, complex with the replicase holoenzyme and, on the other side, comprise a pore through which newly synthesized viral RNA is shuttled into the cytoplasm (9, 11–13). These findings establish nsp3 as a platform for multiple key steps of the replication cycle, including the establishment of the RO, initial RNA replication, viral protein processing, and packaging. Interestingly, in SARS-CoV-2 infection, nsp3 has also been shown to be distributed at low levels throughout the cytoplasm, suggesting it may play additional roles in infection (14).

Within nsp3 reside up to three macrodomains, termed Mac1, Mac2, and Mac3. Macrodomains are a class of conserved protein domains present in all forms of life. Their structure, characterized by a mixed α-β-α sandwich fold, is highly conserved; however, their sequences—and therefore their functions—are diverse (15). Mac2 and Mac3, originally identified in SARS-CoV as part of the SARS-CoV Unique Domain (SUD), are present only in a subset of CoVs, most notably the *Sarbecoviruses*, and were demonstrated to bind nucleic acid (16). Mac1, on the other hand, is highly conserved across coronaviruses and has been shown to be a key virulence factor. Belonging to the MacroD-type class of macrodomains, the CoV Mac1 targets and hydrolyzes ADP-ribose moieties, reversing a post-translational modification known as ADP-ribosylation (17–19).

ADP-ribosylation, the process by which ADP-ribose is covalently attached to target proteins, is facilitated by enzymes known as ADP-ribosyltransferases (ARTs), commonly referred to as PARPs. With 17 members in mammals, the PARP family regulates a myriad of cellular processes, such as stress granule formation, cellular trafficking, DNA damage repair, gene expression, and cell cycle progression (20). Additionally, the importance of PARPs in the innate antiviral response is increasingly clear. Many of the mono-ADP-ribosylating (MARylating) PARPs are IFN-stimulated genes (ISGs) that modulate the inflammatory response and restrict viral replication (21–23). Importantly, ADP-ribose can be added to several different types of residues, including basic and acidic residues, as well as serine and cysteine (24, 25). Importantly, most macrodomains can only remove ADP-ribose from acidic residues but still bind to ADP-ribose attached at other residues. Thus, both ADP-ribose binding and hydrolysis can separately impact biological functions. In CoVs, Mac1 activity is required for IFN repression and efficient viral replication, although the exact targets remain unclear (26–31). Mac1 catalytic activity relies on a highly conserved asparagine (residue N1347 in mouse hepatitis virus [MHV] strain JHM [JHMV]), which coordinates critical hydrogen bonds with the distal ribose (32, 33). Previous characterizations of an alanine mutation at this site that abolished the catalytic activity of Mac1 and sensitized the virus to type-I IFN (IFN-I) signaling, did not affect its ability to bind ADP-ribose (29, 34). In JHMV, an N1347A mutant virus replicated normally in IFN-deficient cells such as 17 Cl-1 but was attenuated in IFN-competent bone marrow–derived macrophages (BMDMs). Importantly, the replication defect was lost in IFNAR knockout cells, demonstrating that Mac1 catalytic activity counters IFN-mediated antiviral responses (34). Soon after, we also generated an alanine mutation at D1329 in JHMV, a residue predicted to coordinate stabilizing interactions with the adenine critical for substrate binding. This mutant demonstrated a large replication defect in both IFN-competent and IFN-deficient cells, suggesting differential roles for the binding and hydrolysis activity of Mac1 (29). Mutation in both residues resulted in a non-replicating virus that was unable to be launched *de novo*. Unsurprisingly, a full in-frame deletion of Mac1 from either JHMV or MERS-CoV resulted in unrecoverable viruses. Interestingly, however, SARS-CoV-2 tolerated the deletion but was sensitive to IFN-γ pre-treatment, similar to the SARS-CoV-2 N1062A mutant virus (31). This result indicates that JHMV and MERS-CoV have a strict requirement for Mac1 ADP-ribose binding, while SARS-CoV-2 may have evolved to encode for a protein with redundant function. We recently found that mutation of a conserved isoleucine in MERS-CoV and SARS-CoV-2 conferred enhanced Mac1 binding affinity to ADP-ribose, with no impact on catalytic activity, but resulted in an overall reduction in viral replication (35). This data together indicates that Mac1 is a critical factor in CoV replication and fitness during infection but relies on finely tuned biochemical properties to promote virus replication and pathogenesis.

In this study, we sought to define the stages of the viral replication cycle reliant on both known biochemical functions of Mac1: binding and catalysis. As predicted, we found that purified SARS-CoV-2 and MERS-CoV D-A mutant proteins demonstrated a stark decrease in substrate affinity, which contrasted with the previously published N-A mutant protein. However, it retained substantial enzymatic activity as opposed to the N-A mutant. Thus, the D-A and N-A Mac1 mutants provide suitable models to better understand the role of ADP-ribose bindings versus hydrolysis in the viral life cycle. In cell culture, the JHMV D1329A mutant virus showed a marked defect in RNA production, while the N1347A mutant virus resembled WT virus in RNA replication kinetics but was significantly reduced for protein accumulation in IFN-competent cells. These results provide a more refined understanding of the multiple mechanisms that Mac1 utilizes to promote CoV replication.

## RESULTS

### Mutation of conserved aspartic acid and asparagine residue to alanine dramatically reduces ADP-ribose binding but maintains ADP-ribosylhydrolase activity

**Fig 1:**
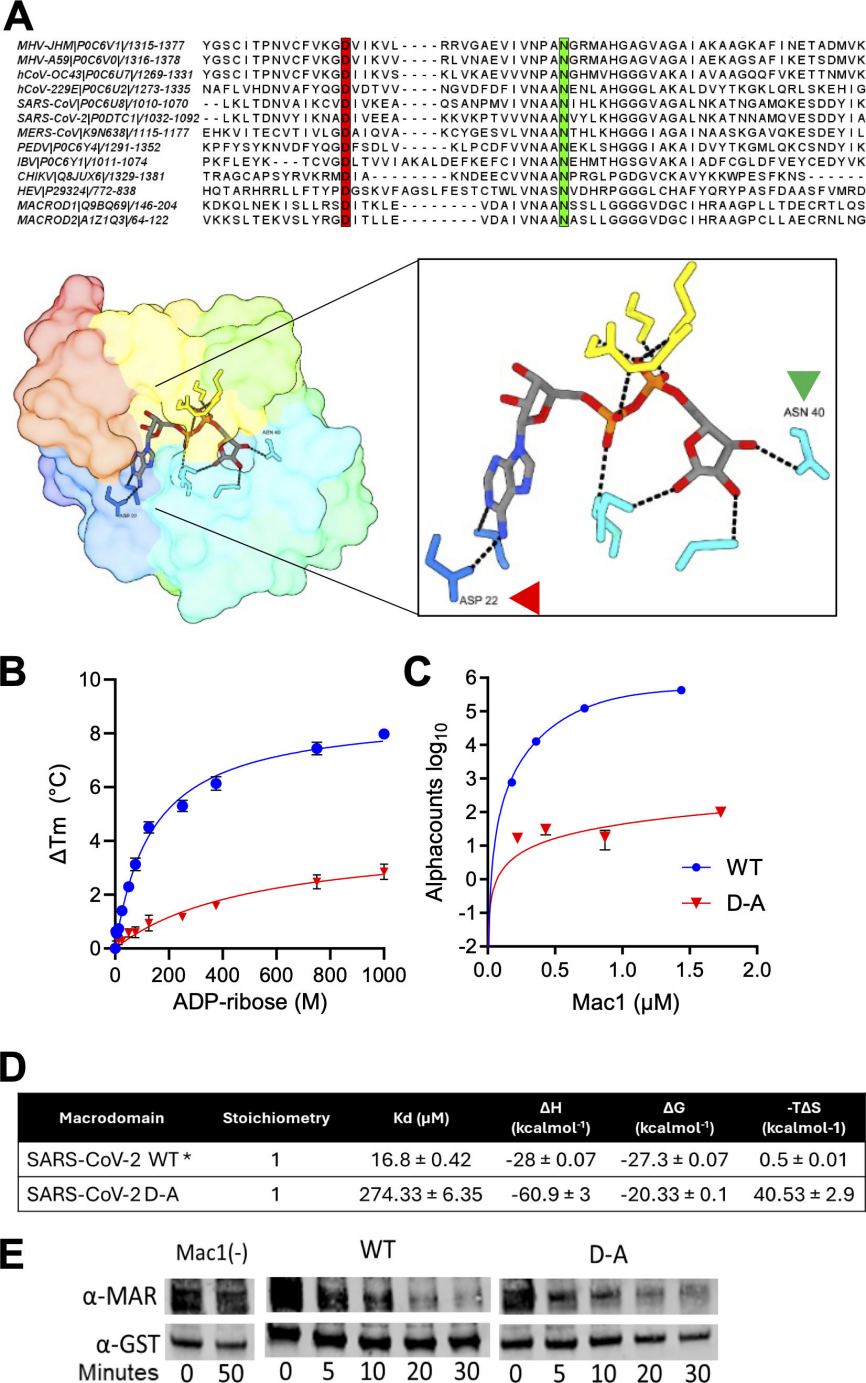
Mutation of a highly conserved aspartic acid in the ADP-ribose binding pocket of Mac1 dramatically reduces ADP-ribose binding but largely maintains its enzyme activity. (**A**) Highly conserved aspartic acid (red) and asparagine (green) residues highlighted in alignments of coronavirus Mac1 and other related macrodomain proteins. (**B**) SARS-CoV-2 Mac1-WT and D-A proteins were incubated with increasing concentrations of ADP-ribose and then assayed for thermal stability by differential scanning fluorimetry (DSF). (**C**) SARS-CoV-2 Mac1 WT and D-A proteins were tested for their ability to interact with an ADP-ribosylated peptide using an AlphaScreen assay. The results in B and C are representative of two independent experiments. (**D**) Isothermal titration calorimetry (ITC) of purified SARS-CoV-2 WT and D-A proteins in the presence of increasing concentrations of ADP-ribose. *SARS-CoV-2 WT protein was previously published (27). The results are the combined averages of four independent experiments. (**E**) WT and D-A proteins were incubated with MARylated GST-PARP10 CD for the indicated time, and MARylation levels were measured by Western blotting for mono-ADP-ribose. GST antibody was used as a loading control. Results are from one experiment representative of two independent experiments.

Two highly conserved residues in JHMV Mac1, N1347 and D1329 (Fig. 1A), were previously demonstrated to impact viral fitness and virulence, although to different degrees and in a cell type–specific manner (29). To compare the biochemical properties of the D-A mutation with the previously characterized N-A mutation (29, 35), we performed several previously established ADP-ribose binding and hydrolysis assays using purified SARS-CoV-2 and MERS-CoV Mac1 WT and D-A proteins. We and others have been unable to produce MHV Mac1 protein, so SARS-CoV-2 and MERS-CoV proteins were used instead. ADP-ribose binding assays include a differential scanning fluorimetry (DSF) assay that measures thermal stability, a proximity-based AlphaScreen assay, and isothermal calorimetry (ITC), which measures the thermodynamic properties of binding. Thermal stability of the WT-Mac1 protein was demonstrated to be approximately 6°C higher than that of the D-A protein at the endpoint concentration of 1 M ADP-ribose (Fig. 1B). In the AlphaScreen assay, the D-A protein had a multi-log reduction in alpha counts compared to the WT protein (Fig. 1C). Finally, isothermal titration calorimetry (ITC) data showed that the *K_D_* of Mac1 D-A to ADP-ribose was ~16-fold higher than the WT–ADP-ribose interaction (Fig. 1D). We also saw similar results for each of these assays using MERS-CoV Mac1 WT and D-A proteins (Fig. S1). These data together demonstrate a marked defect in the Mac1 D-A protein’s ability to bind ADP-ribose and distinguish the D-A mutant from the N-A mutant, which was previously shown to bind ADP-ribose at near-WT levels (35). In addition, we performed an *in vitro* ADP-ribosylhydrolase assay by co-incubating purified SARS-CoV-2 Mac1-WT and D-A proteins with mono-ADP-ribosylated (MARylated) PARP10 and measured the loss of MAR signal from PARP10 by Western blotting, as previously described (27). We found that Mac1-D-A retained substantial enzyme activity, which contrasts with Mac1-N-A, which we previously found to be largely devoid of catalytic activity (Fig. 1E) (35) These results are consistent with results from alphavirus macrodomain studies. These biochemical studies provide a stark delineation between the D-A and N-A mutant proteins as being deficient in either binding or hydrolysis, respectively.

### MHV D1329A/D1330A virus is attenuated in cell culture and *in vivo*

**Fig 2:**
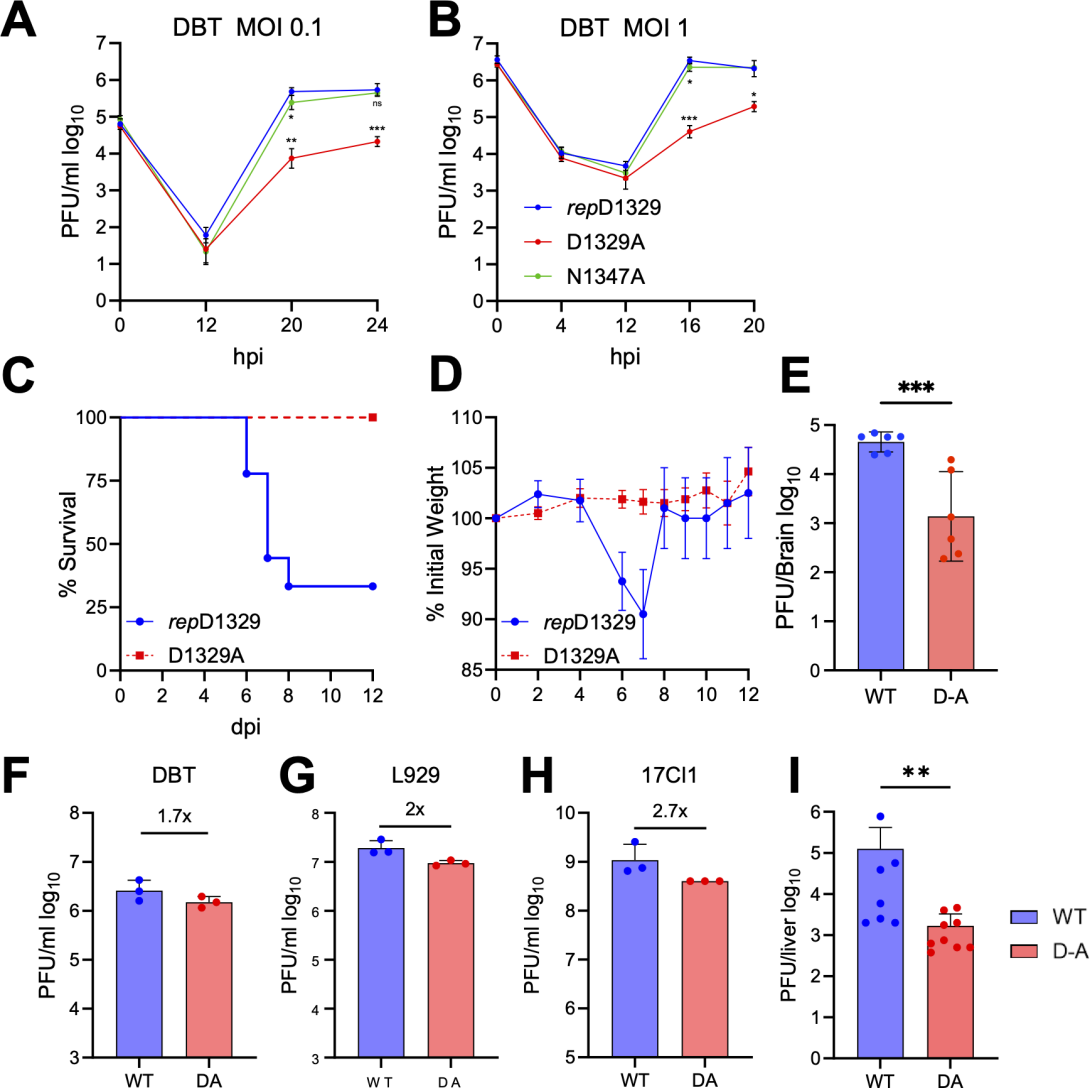
MHV-D1329A/D1330A virus is attenuated in cell culture and *in vivo*. (**A–B**) DBT cells were infected with JHMV-*rep*D1329, N1347A, and D1329A viruses at an MOI of 0.1 (**A**) and 1.0 (**B**). Progeny virus was collected at indicated times and was titered by plaque assay. The results are from a single experiment representative of two independent experiments. (**C–D**) C57BL/6 mice were infected with 10^4^ PFU JHMV-*rep*D1329 and D1329A virus and monitored for survival (**C**) and weight loss (**D**) over 12 days. *n* = 9 mice, *rep*D1329; *n* = 10 mice, D1329A. The results are the combined data from two independent experiments. (**E**) C57BL/6 mice were infected intranasally with 10^4^ PFU JHMV-*rep*D1329 and D1329A virus. Brains were harvested at six dpi, and viral loads were measured by plaque assay. *n* = 6 mice per group. (**F–I**) DBT (**E**), L929 (**F**), and 17 Cl-1 (**G**) cells were infected with MHV-A59 WT and D1330A viruses, and progeny virus was measured at 24 hpi. The results are from a single experiment representative of two independent experiments. (**H**) C57BL/6 mice were infected with 500 PFU A59-WT and D1330A viruses. Livers were collected at three dpi in PBS, and viral loads were measured by plaque assay. Graphs represent the geometric mean with 95% confidence interval. *n* = 7 mice, WT; *n* = 9 mice, D1330A.

Previous work with the JHMV single-nucleotide D1329A mutant virus revealed a high propensity for reversion *in vivo*, indicating a high fitness cost compared to other viable Mac1 mutants (29). To prevent this reversion, the JHMV-D1329A mutant virus was regenerated to contain a two-nucleotide substitution using a bacterial artificial chromosome (BAC) based reverse genetic system (36). We produced this virus in cell culture and confirmed that it had no reverting mutations. Furthermore, to confirm that the defect is not due to second-site mutations produced during cloning, we reintroduced the WT Mac1 sequence into the JHMV-D1329A BAC which was termed the “repaired” D1329A (*rep*D1329). In addition, we fully sequenced the MHV genome from terminal passages of D1329A and found no additional mutations in the viral genome. At low (0.1) and high (1.0) MOI, this new JHMV-D1329A mutant virus demonstrated the same multi-log replication defect in DBTs when compared to *rep*D1329 and N1347A viruses, as previously described (Fig. 2A and B) (29). Importantly, intranasal infection of C57BL/6 with JHMV-D1329A resulted in no lethality or weight loss compared to infection with *rep*D1329, which resulted in ~25% mouse survival and an average of more than 10% wt loss (Fig. 2C and D). In addition, D1329A had, on average, more than 1-log lower viral loads in the brains of infected mice at six days post-infection (dpi) (Fig. 2E). These results indicate that this two-base pair mutant D1329A virus, unlike the single-base pair mutant, could not readily revert to WT *in vivo*. We also engineered the orthologous mutation into MHV-A59 (D1330A). The A59-D1330A virus had a mild replication defect in cell culture (Fig. 2F through H) but replicated poorly *in vivo* compared to WT virus (Fig. 2I), indicating that Mac1 ADP-ribose binding is critical across multiple strains of MHV.

### JHMV-D1329A produces small plaques and demonstrates reduced infectivity in mouse cells compared to *rep*D1329 and N1347A viruses

**Fig 3:**
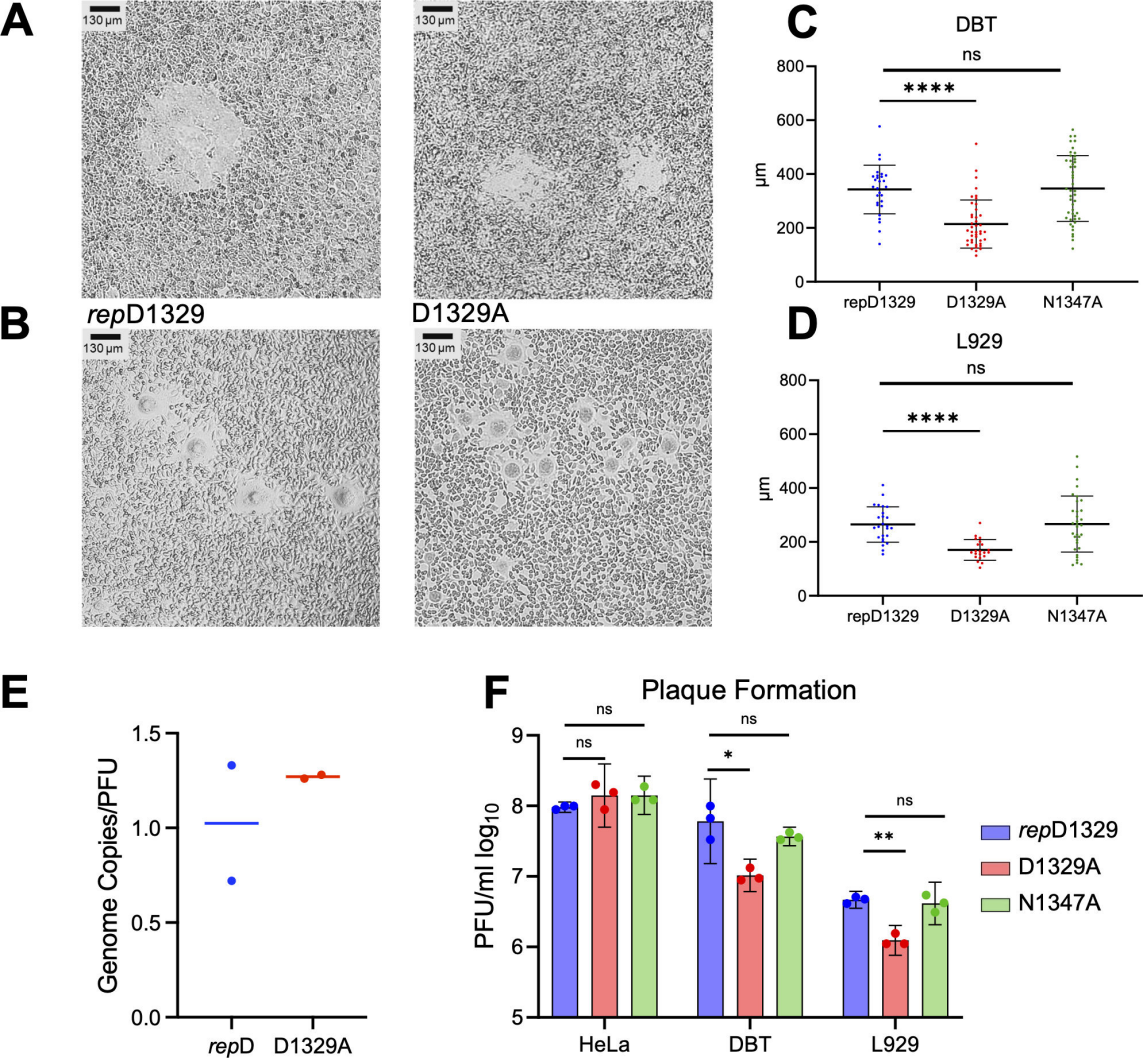
JHMV-D1329A virus produces small plaques and demonstrates reduced infectivity in mouse cells compared to *rep*D1329 and N1347A viruses. (**A–B**) DBT (**A**) and L929 (**B**) cells were infected with JHMV-*rep*D1329 and D1329A at an MOI of 0.1 and were examined at 18 (DBT) or 20 (L929) hpi to determine relative plaque sizes between mutants. (**C**) Plaque sizes were measured using CaptaVision software calibrated with a stage micrometer *n* = 27, *rep*D1329; *n* = 41, D1329A; *n* = 45, N1347A. (**D**) Plaque sizes were measured using CaptaVision software calibrated with a stage micrometer *n* = 25, *rep*D1329; *n* = 21, D1329A; *n* = 33, N1347A. The results represent the combined results of two independent experiments. (**E**) Genome copy was determined using cDNA derived from viral stocks and measured using qPCR with primers as described in methods. Copy number was determined by comparing the Ct values of the virus stocks to a standard curve generated using MHV BAC DNA. The genome-to-PFU ratio was then determined by dividing the genome copy by the viral titer. (**F**) HeLa-MVR, DBT, and L929 cells were infected with equal PFUs of each virus, and the number of plaques was counted 20 hpi. The results are from a single experiment representative of three independent experiments.

The first notable hallmark of the JHMV-D1329A virus infection was a substantial reduction in overall plaque size in both DBT and L929 cells (Fig. 3A and B). We quantified the size of D1329A plaques compared to *rep*D1329 and N1347A viruses and found that the D1329A plaques were, on average, about half the size of the plaques produced by *rep*D1329 and N1347A on both DBT and L929 cells (Fig. 3C and D). JHMV plaques are formed by cell-cell syncytia mediated by the S protein (37). Thus, this phenotype is likely due to a severe reduction in the production of S protein or its ability to traffic to the cell surface. A severe lack of S protein production could be due to the inability to initiate infection in some cells. To determine if JHMV-D1329A has a defect in initiating a productive infection, we performed a plaquing efficiency assay using DBT, L929, and HeLa cells expressing the MHV receptor (HeLa-MVR). First, we compared the genome-to-PFU (gRNA/PFU) ratio for the D1329A virus compared to *rep*D1329. We found that the gRNA/PFU ratio of the D1329A virus was not reduced when compared to *rep*D1329 (Fig. 3E), which we also previously showed for the N1347A virus (38). As the gRNA/PFU ratios were similar, we plated an equal number of plaque-forming units (PFU) of *rep*D1329, D1329A, and N1347A on each cell type, and the number of plaques was determined. D1329A produced ~5- to 10-fold fewer plaques on both DBT and L929 cells compared to *rep*D1329 and N1347A, indicating that D1329A has a substantial defect in the ability to initiate a productive infection on mouse cells (Fig. 3F).

### JHMV-D1329A has no defect in genomic RNA delivery into cells

**Fig 4:**
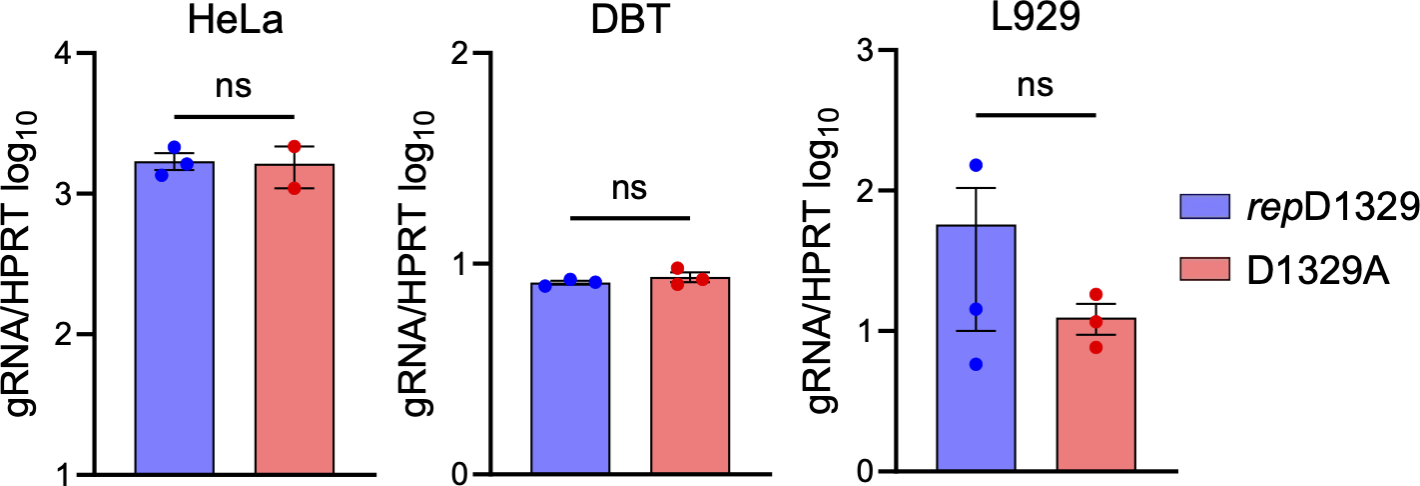
JHMV-D1329A virus has no defect in genomic RNA delivery into cells. Indicated cell lines were infected with JHMV *rep*D1329 and D1329A viruses for one hour, then trypsinized for five minutes, and rinsed three times with PBS. The cells were then collected, RNA was harvested, and genomic RNA levels were determined by qPCR using the ΔCt method and normalized to HPRT mRNA. These data are from a single experiment representative of two independent experiments.

To determine if the inability of the D1329A virus to initiate infection stems from a reduction in the entry of viral RNA into the cytoplasm of infected cells, we measured intracellular genomic RNA levels at two hours post-infection (hpi) via RT-qPCR. Each of the three indicated cell lines was infected with JHMV-*rep*D1329 and D1329A for two hours; after which, the cells were trypsinized and washed three times with PBS to remove non-infective virions and then resuspended in TRIzol Reagent (Invitrogen). RT-qPCR revealed no significant reduction in D1329A gRNA levels when compared to *rep*D1329, suggesting that the plaquing defect is not due to a defect in viral entry (Fig. 4).

### JHMV-D1329A virus is defective in genomic and subgenomic RNA accumulation compared to *rep*D1329 and N1347A viruses

**Fig 5:**
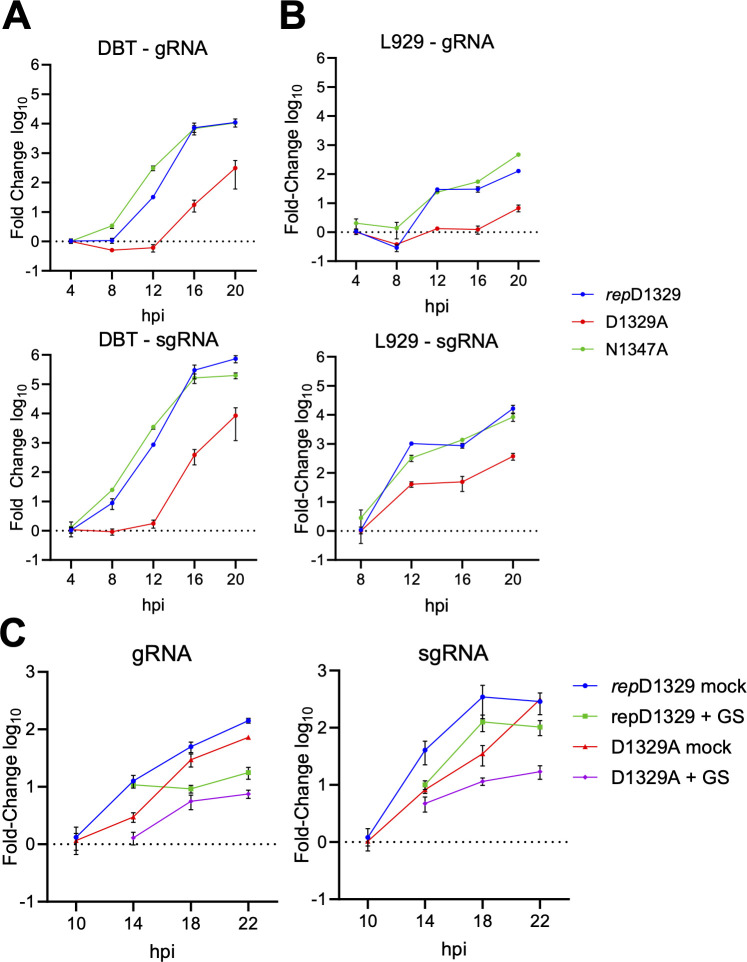
JHMV-D1329A virus is defective in the production of viral genomic RNA (gRNA) and subgenomic RNA6 (sgRNA6) compared to *rep*D1329 and N1347A viruses. (**A–B**) DBT (**A**) and L929 (**B**) cells were infected with indicated viruses at an MOI of 3.0. Cells were collected at indicated times, and gRNA (top) and sgRNA6 (bottom) were measured by qPCR using the ΔΔCT method and normalized to HPRT. The data in A and B are from a single experiment representative of two independent experiments. (**C**) L929 cells were infected with either *rep*D1329 or D1329A viruses at an MOI of 0.1. At 10 hpi, cells were treated with Remdesivir metabolite GS-441524 or DMSO control. Cells were collected at indicated time points, and viral gRNA (left) or sgRNA6 (right) levels were determined by qPCR using the ΔΔCt method and normalized to HPRT mRNA. The results are from a single experiment representative of two independent experiments.

Next, we assayed the accumulation of viral genomic and subgenomic RNAs in both high and low MOI infections of DBT and L929 cells. Using RT-qPCR, viral genomic RNA (gRNA) and subgenomic RNA6 (sgRNA6) levels were quantified over a 20-hour period (Fig. 5A and B). We used sgRNA6 as a marker for subgenomic RNAs as the primers for sgRNA6 gave the most robust results in our qPCR assay. At high MOI, the N1347A virus closely resembled the *rep*D1329 virus, as viral sgRNA6 and gRNA levels increased substantially between four and 12 hpi in DBTs, while D1329A-infected cells failed to produce any new viral transcripts before 12 hpi. This difference was most notable in sgRNA6, as *rep*D1329 and N1347A transcripts were increased by nearly 3 logs over the D1329A virus. D1329A-infected cells did show increasing RNA production after 12 hpi, indicating a delay in transcription initiation, although the levels never reached that of *rep*D1329- or N1347A-infected cells (Fig. 5A). Similarly, infection of L929s showed approximately 10- to 100-fold increases in gRNA and sgRNA6 levels in the *rep*D1329- and N1347A-infected cells compared to D1329A throughout the course of infection (Fig. 5B). Similar results were seen at low MOI (Fig. S2). To determine whether the defect in D1329A RNA accumulation was due to targeted degradation of viral transcripts, infected L929 cells were treated with the Remdesivir metabolite GS-441524 at 10 hpi to arrest RdRp-mediated transcription (39). Viral transcripts were quantified at four-hour intervals until 22 hpi (Fig. 5C). GS-441524 was not added until 10 hpi, as earlier addition may have limited the amount of RNA produced during D1329A infection, making it unfeasible to detect RNA degradation. Viral RNA synthesis increased in GS-441524–treated cells for a few hours, after which RNA levels stabilized, whereas RNA levels in untreated cells continued to increase. In both the *rep*D1329 and D1329A infections treated with GS-441524, there was no evidence of RNA degradation as RNA levels did not significantly decrease through the course of the infection, indicating that there is no increased degradation of the D1329A transcripts compared to *rep*D1329.

**Fig 6:**
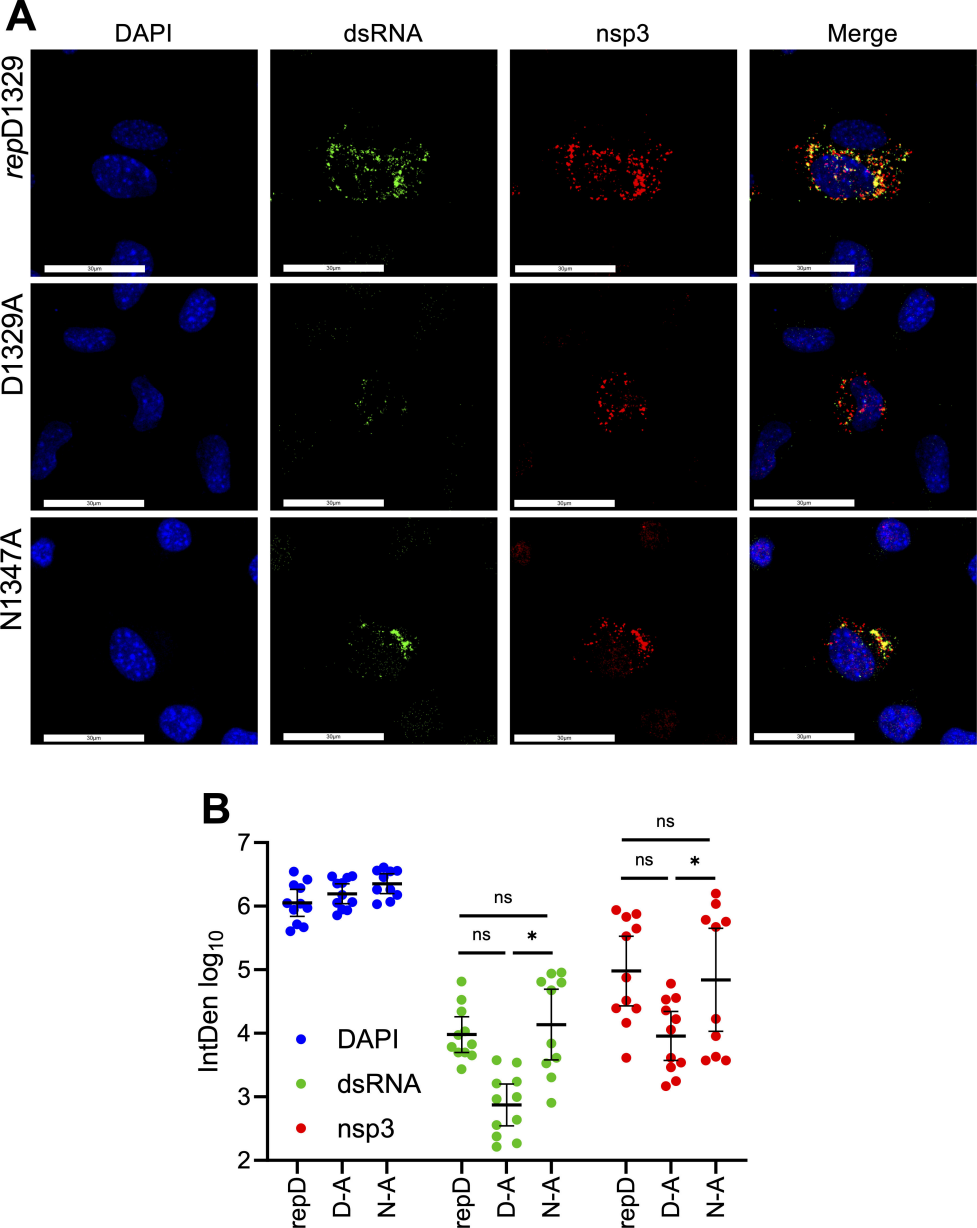
JHMV-D1329A virus has reduced dsRNA levels early in infection. (**A**) DBTs were infected with indicated virus at an MOI of 0.5, fixed at eight hpi, stained for dsRNA and nsp3, and analyzed by confocal microscopy. (**B**) Quantification of fluorescence signal displayed as a product of mean pixel intensity and thresholded area (IntDen). Error bars indicate geometric mean with 95% CI. Data are combined data from two separate experiments. Statistics for 6B was done using an ordinary one-way ANOVA.

As a separate method to analyze viral RNA production, we performed confocal immunofluorescence microscopy using probes for dsRNA, a hallmark RNA replication intermediate (Fig. 6A). DBT cells were infected with *rep*D1329, D1329A, and N1347A at an MOI of 0.5, and cells were fixed and stained for nsp3 and dsRNA at eight hpi. While mock-infected cells were devoid of dsRNA signal, *rep*D1329- and N1347A-infected cells demonstrated robust dsRNA staining that co-localized with nsp3, indicating that the dsRNA represented viral RNAs. In contrast, D1329A-infected cells had minimal dsRNA despite having significant nsp3 staining. Quantitation of these images confirmed that the D1329A virus produced substantially reduced levels of dsRNA, but only mildly reduced levels of nsp3, with several images showing overlapping in signal intensities (Fig. 6B). This result indicates that D1329A-infected cells produced nsp3 but were unable to efficiently produce RNA. N1347A, conversely, appeared to produce similar levels of nsp3 and dsRNA staining compared to *rep*D1329. To further determine if D1329A can efficiently translate non-structural proteins, we stained for nsp3 at six hpi, the earliest time point at which it could be detected. Imaging demonstrated that D1329A produced nsp3 at these early time points at levels that were reduced, but not significantly different from *rep*D1329 and N1347A (Fig. S3A and B). The reduction in nsp3 is likely due to already reduced RNA production in these cells, as more genomic RNA would lead to increased production of nsps for the *rep*D1329 or N1347A viruses. In combination, these results suggest that Mac1 ADP-ribose binding is required for efficient viral transcription initiation in murine cells, whereas hydrolysis activity is likely dispensable in the earliest stages of replication.

### N1347A infected primary bone marrow–derived macrophages (BMDMs) produce WT levels of viral RNA but have reduced N protein accumulation compared to *rep*D1329 virus

**Fig 7:**
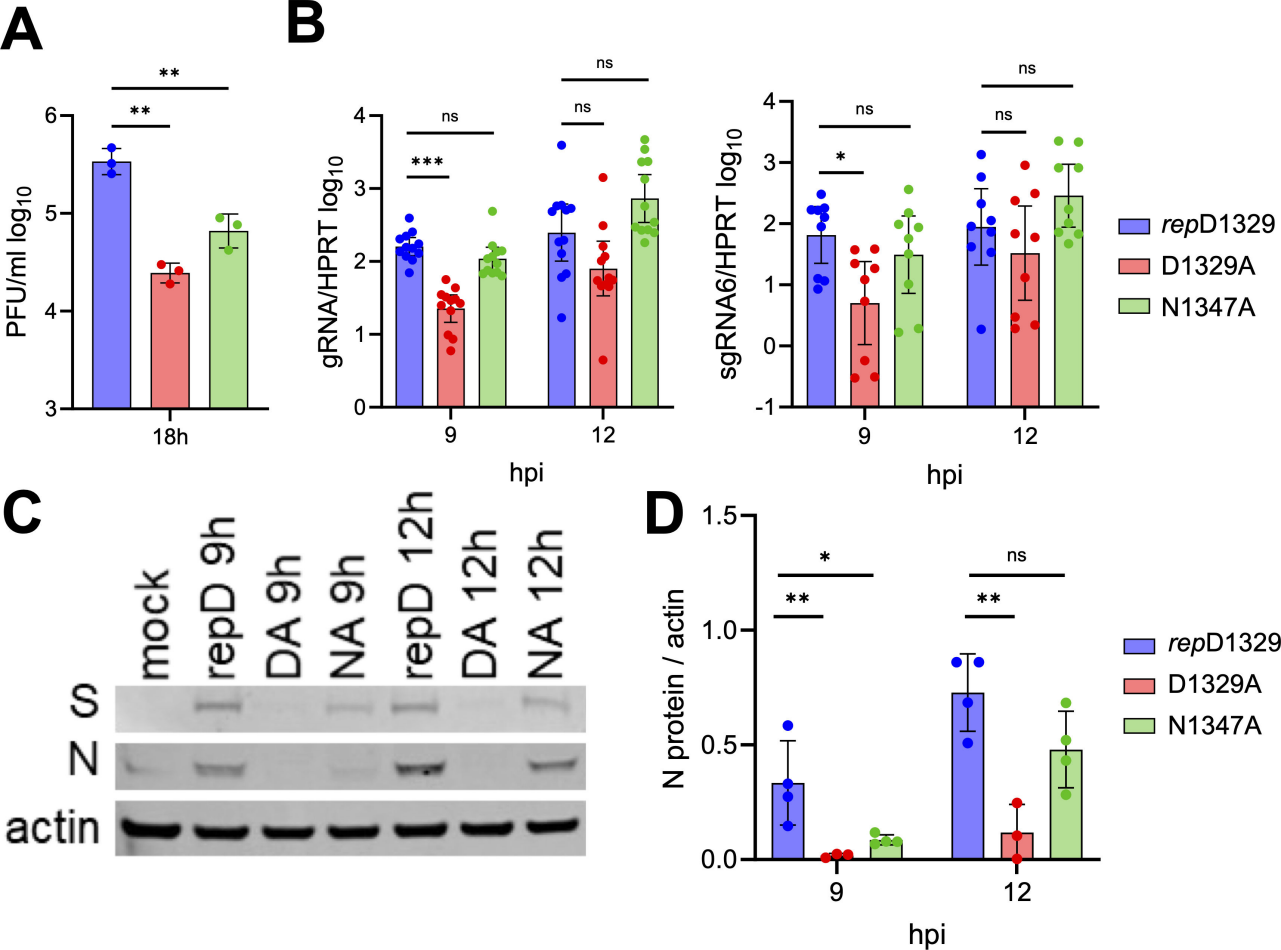
N1347A-infected BMDMs produce WT levels of viral RNA but have reduced N protein accumulation compared to *rep*D1329 virus. (**A**) M2-differentiated BMDMs were infected with indicated viruses at an MOI of 1.5. Cells and supernatants were collected at 18 hpi, and progeny virus was measured by plaque assay. The results are from a single experiment representative of two independent experiments. (**B**) BMDMs were infected as in (**A**), and cells were collected at indicated time points, and gRNA (left) and sgRNA6 (right) were measured by qPCR using the ΔCt method and normalized to HPRT mRNA. The data in B represent the combined results of three (sgRNA6) or four (gRNA) independent experiments. (**C**) BMDMs were infected as in (**A**), and cells were collected in sample buffer at indicated time points and viral spike (**S**) and nucleocapsid (**N**) proteins were analyzed by Western blotting. Actin was used as a loading control. The results are from a single experiment representative of two to four independent experiments. (**D**) Quantitation of the Western blot signal in C normalized to actin using Image J. The data in (**D**) represent the combined results from our (*rep*D1239 & N1347A) or three (D1329A) independent experiments.

We previously demonstrated that both N1347A and D1329A mutants have similar replication defects in primary macrophages when infected at low MOI, which could be partially rescued by treatment with PARP inhibitors (29). However, N1347A replication could also be rescued in IFNAR and PARP12 knockout cells, while D1329A replication was not, indicating that these viruses may impact different parts of the viral life cycle in these cells (31, 34). First, we tested whether these viruses had replication defects at high MOI, allowing us to evaluate the viral life cycle within a single cycle of replication. As expected, both viruses had replication defects in BMDMs when infected at an MOI of 1.5 (Fig. 7A). Specifically, D1329A virus titers were reduced almost 14-fold from *rep*D1329, while N1347A titers were reduced 5-fold. Next, we evaluated the accumulation of viral gRNA and sgRNA6. D1329A infection resulted in a significant reduction in both gRNA and sgRNA6 levels at nine hpi, while gRNA and sgRNA6 levels following N1374A infection were similar to those of *rep*D1329, as seen in both DBT and L929 cells (Fig. 7B). However, when we evaluated the production of viral structural proteins, N1347A-infected cells produced reduced levels of nucleocapsid (N) and spike (S) proteins at nine and 12 hpi (Fig. 7C). When quantitating viral protein levels from multiple experiments, the amount of N protein was significantly reduced almost 4-fold at nine hpi compared to *rep*D1329 infection, while at 12 hpi, N protein was reduced 1.5-fold (Fig. 7D).

**Fig 8:**
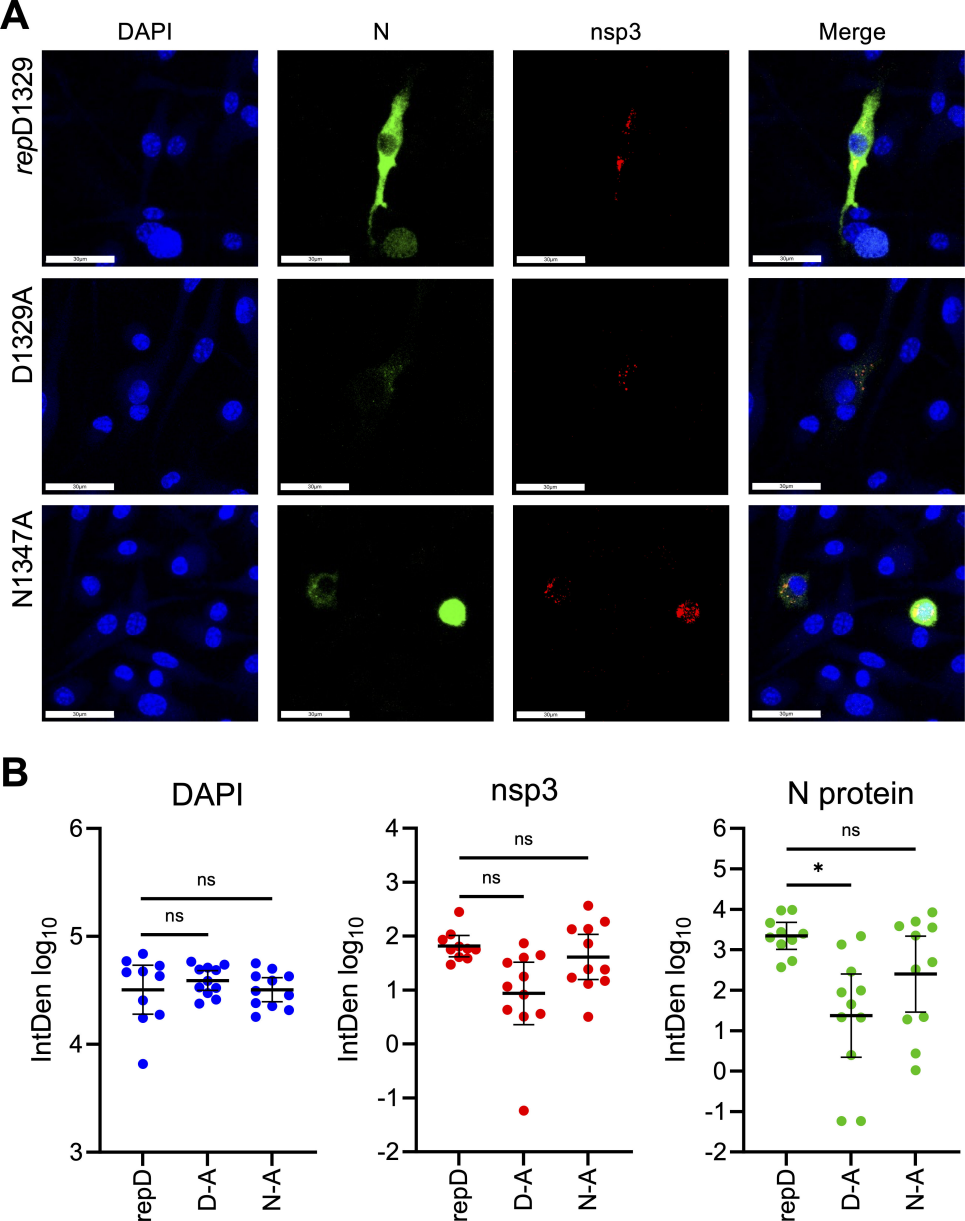
D1329A- and N1347A-infected BMDMs have near *rep*D1329 levels of nsp3 but have reduced N protein abundance. (**A**) BMDMs were infected with indicated virus at an MOI of 0.5 and fixed at nine hpi, stained for nsp3 and N protein, and analyzed by confocal microscopy. (**B**) Quantification of fluorescence signal displayed as a product of mean pixel intensity and thresholded area (IntDen) for all three fluorescent channels. Combined data from two independent experiments presented as geometric mean with 95% confidence intervals.

 Additionally, we evaluated nsp3 and N protein abundance by confocal immunofluorescence in BMDMs. BMDMs were infected at an MOI of 0.5, followed by fixation and staining for nsp3 and N protein at nine hpi (Fig. 8A). Nsp3 puncta were again observed across all three viruses. Quantification showed that the D1329A-infected cells had 7.5-fold less nsp3 staining compared to *rep*D1329, while nsp3 levels were only reduced 1.6-fold in N1347A-infected cells, neither of which was significant (Fig. 8B, middle). In fact, in several cells infected with D1329A, nsp3 levels were at or just below the mean of the *rep*D1329-infected cells, again demonstrating that D1329A can produce sufficient levels of nsp3 early in infection. This contrasts with N protein staining, which was reduced nearly 100-fold in D1329A-infected cells and almost 10-fold in N1347A-infected cells (Fig. 8B, right). However, the level of N protein staining in N1347A-infected cells was highly variable within individual cells, with some cells producing normal levels of N protein, while others were dramatically reduced. As N1347A is restricted by IFN-induced PARPs (34), these results suggest that PARP induction levels between individual cells may fluctuate, resulting in inconsistent reductions in N protein production compared to *rep*D1329. But in general, N1347A-infected cells produced less N protein, but not nsp3, than the *rep*D1329-infected cells. These observations suggest that initial nsp3 synthesis is not substantially impacted by Mac1, but that subsequent viral RNA and late protein production rely on the binding and hydrolysis activities, respectively, of Mac1 to proceed efficiently.

## DISCUSSION

The current understanding of the conserved CoV macrodomain relies largely on its characterization as an ADP-ribosylhydrolase. Previous work has highlighted the importance of PARP activity and Mac1’s de-ADP-ribosylating activity on viral fitness and pathogenesis in both coronaviruses and alphaviruses (40). We have previously generated alanine mutations in several conserved residues of the Mac1 binding pocket. We found that mutation of a conserved asparagine residue important for hydrolysis activity, N1347A in JHMV, attenuated the virus in primary macrophages and sensitized it to PARP activity. We also found that mutation of another conserved residue, D1329, predicted to coordinate binding affinity, conferred a greater replication defect for this virus. Finally, we have been unable to recover JHMV that incorporates both mutations or a full Mac1 deletion. These findings led us to hypothesize that Mac1 is critical for JHMV infection and serves multiple roles in the viral replication cycle.

 In this study, we sought to determine the stages of the viral life cycle most reliant on the binding and hydrolysis activities of Mac1, in an effort to better understand the roles Mac1 plays in infection. To characterize the biochemical impacts of the conserved aspartic acid and asparagine residues in the ADP-ribose binding pocket, we utilized purified SARS-CoV-2 and MERS-CoV Mac1 proteins with each mutated to alanine and then performed *in vitro* ADP-ribose binding and hydrolysis assays. These data demonstrated a stark delineation between these mutant Mac1 proteins and their ADP-ribose binding and hydrolysis activities. Previous work from our group established that the N-A mutant Mac1 exhibited little to no loss in substrate binding affinity but had greatly reduced hydrolysis activity (35), while here we found that the D-A mutant Mac1 showed a multi-log reduction in binding affinity, but only a slight reduction in hydrolysis. Importantly, similar results were demonstrated by other groups with SARS-CoV-2 mutant proteins and with recombinant alphavirus macrodomain proteins as well (33, 41). Next, we sought to compare the impact of both mutations on viral replication in the early viral life cycle. To achieve this, we utilized the previously generated JHMV-N1347A virus and re-generated a new clone of the previously studied D1329A virus. This new clone was generated via a two-base pair substitution in the D1329 codon, rather than one to reduce the likelihood of reversion. As expected, this virus demonstrated severe attenuation in cell culture and *in vivo*. Through the detection of dsRNA by confocal microscopy and specific determination of viral genomic and subgenomic RNA levels by qPCR, we found that the D1329A virus does not efficiently initiate viral RNA production following infection of any murine cell line that is susceptible to JHMV (Fig. 5 and 6). Furthermore, the D1329A virus produced similar amounts of nsp3 in BMDMs as the *rep*D1329 virus, and puncta of nsp3 staining in D1329A-infected cells often lacked any notable dsRNA staining. In contrast, the N1347A virus replicated viral RNA at levels equal to that of WT virus in all cell lines tested but had a notable defect in viral protein production in primary BMDMs (Fig. 7). These data indicate that the binding activity of Mac1 is key for early RNA production, while the hydrolysis activity is important for accumulation of viral proteins later on in the replication cycle. However, we cannot rule out the possibility that other defects, such as in polyprotein processing or assembly, may also be present. In fact, considering that N protein levels in N1347A-infected BMDMs were almost back to WT levels at 12 hpi, it is likely that there may be further defects in the assembly process for this virus. Below, we present several hypotheses for how each activity may promote replication.

### ADP-ribosylhydrolase activity

We have demonstrated that PARP12 and PARP14 are both required to restrict the replication of CoVs with catalytically inactive Mac1 (21, 31, 42). Others have found that PARP14 is required for the production of ADP-ribose puncta following IFNγ or poly(I:C) treatment (34). Within these puncta, both PARP14 and DTX3L, an E3 ubiquitin ligase, are likely ADP-ribosylated, with DTX3L/PARP9 regulating the activity of PARP14 (43, 44). As an E3 ubiquitin ligase, it is possible that DTX3L ADP-ribosylation enhances its ability to target viral proteins for degradation. However, there are many other potential targets of PARP14, such as p62, an autophagy regulator, and RACK1, a ribosome binding protein, which could impact viral protein production through autophagic degradation or repression of translation, respectively (45, 46). Furthermore, PARP12 is localized to the Golgi, and cell stress can trigger PARP12 movement from the Golgi and a block in anterograde-membrane trafficking, which could impact viral assembly (47). Further work is required to decipher which PARP12/14 target(s) restrict viral protein production or assembly following infection with Mac1 catalytic-mutant CoVs.

### ADP-ribose binding activity

**Fig 9:**
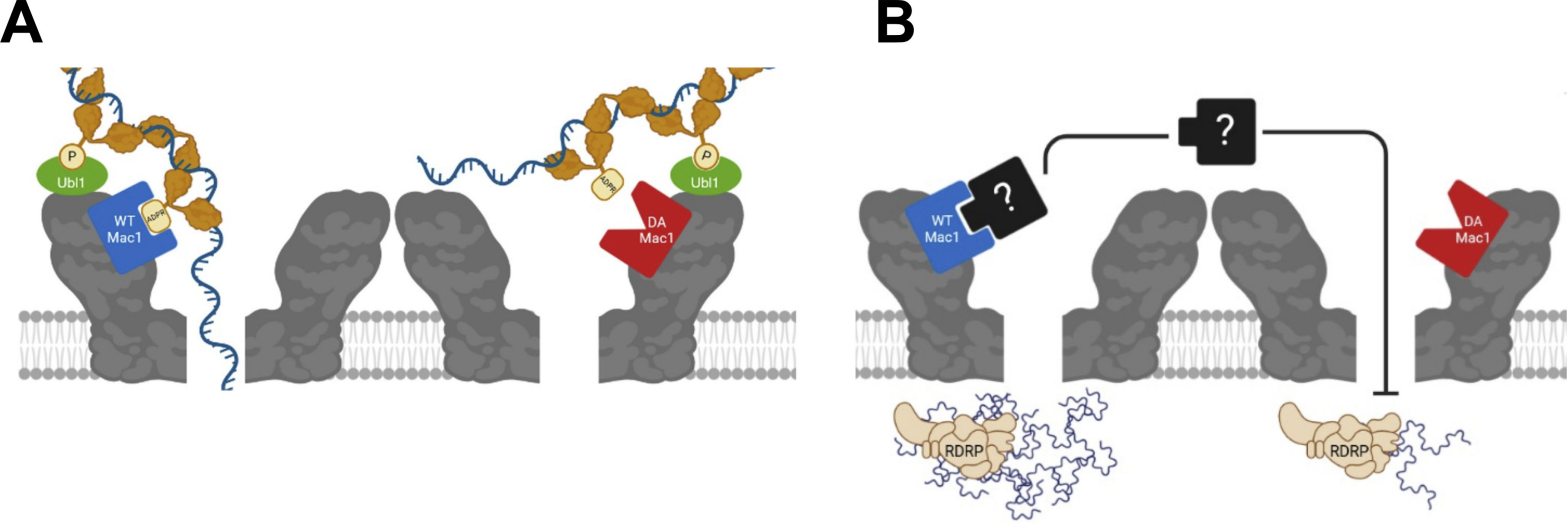
Potential roles for Mac1 in regulating early RNA transcription. (**A**) The Ubl1 domain of nsp3 interacts with phosphorylated residues on the RNA-bound nucleocapsid protein, which is a key factor in shuttling viral RNA through the nsp3 pore to the site of transcription. Mac1 binding affinity may enhance this interaction between nsp3 and N protein, without which there is a delay in transcription initiation. (**B**) Mac1 binding affinity may sequester or block ADP-ribosylated host factors that target and inhibit viral transcription early in the replication cycle.

We envision two separate but not mutually exclusive hypotheses for how this Mac1 function promotes viral RNA production. Recent work has demonstrated that nsp3 plays a major role in forming a hexameric complex that constitutes a pore in the replication organelle, through which viral RNA is thought to pass (9, 11). As part of the larger pore structure, the N-terminal domains of nsp3 extend into the cytoplasm, where one or more subunits may interact with a number of host and viral factors. Previous work has established the association of nsp3 with the nucleocapsid protein as a critical factor in establishing CoV infection (8, 48, 49). Current models postulate that, since CoV gRNA serves as both the initial mRNA for nsp translation and the template for RNA replication, N protein may shuttle gRNA into the replication organelle via the nsp3 pore (8). Mutational analyses from several groups have demonstrated that this process largely relies on the interaction of the N-terminal Ubl1 of nsp3 with multiple phosphorylated residues in the serine- and arginine-rich regions N1b and N2a of N protein (48–50). Other characterizations of the CoV nucleocapsid have revealed that it is also ADP-ribosylated (51). From the current data, it appears that the N protein is ADP-ribosylated only during infection, and the modification is retained in the virion. Moreover, the modification was shown to be unaffected by catalytically active Mac1. Thus, in our first hypothetical model, Mac1 would interact with an ADP-ribosylated N protein to promote the translocation of either the viral genome or N protein through the pore (Fig. 9A). Since N protein phosphorylation is reliant on host kinases (52), and ADP-ribosylation may compete with phosphorylation on some residues, Mac1 binding to ADP-ribosylated N may have evolved as an alternative measure to maintain the interaction between N protein and nsp3. Identification of the sites of N protein ADP-ribosylation and mutation analysis would help clarify how ADP-ribosylation affects N protein’s ability to interact with nsp3.

 However, the hypothesis best supported by the current data is that the ADP-ribose binding affinity of Mac1 serves to prevent a key antiviral host factor from disrupting the initiation of viral transcription. Plaquing efficiency assays from this study showed that the D1329A mutant virus more readily establishes productive infections in HeLa-MVR cells than in murine DBT or L929 cells, suggesting a host-dependent antiviral response. Furthermore, treatment with PARP inhibitors partially rescues D1329A virus replication, and treatment with the PARP substrate NAD^+^ further reduces the replication of the D1329A virus (29). These results in combination suggest that there is a murine-specific host factor that restricts virus replication in an ADP-ribosylation-dependent manner. Binding of Mac1 to an unknown ADP-ribosylated host factor (or factors) may prevent it from traversing the nsp3 pore to access the replication-transcription complex (RTC) and thereby repress viral RNA production (Fig. 9B). Structural analysis of the pore complex predicts a central channel of around 17 Å (53) that may be large enough for small proteins to access the RTC. Previous studies of related alphavirus macrodomain mutant viruses found that macrodomain/ADP-ribose binding is also required for initiating efficient viral RNA synthesis, indicating that there may be a conserved host mechanism that represses the initial transcription of multiple viruses (41, 54). Thus, future research into these mechanisms could identify novel methods for developing antiviral therapeutics targeting multiple classes of RNA viruses.

## MATERIALS AND METHODS

### Cell culture

Delayed Brain Tumor (DBT), L929, 17 CL-1, and HeLa cells expressing the MHV receptor CEACAM1 (HeLa-MVR) were cultured in Dulbecco’s Modified Eagle Medium (Corning) supplemented with 10% fetal bovine serum, 100 U/mL penicillin, 100 mg/mL streptomycin, 10 mM HEPES, 1 mM sodium pyruvate, 0.1 mM non-essential amino acids, and 2 mM L-glutamine. Bone marrow–derived macrophages (BMDMs) were obtained from WT C57BL/6 mice and differentiated into M0 macrophages by culturing for seven days in Roswell Park Memorial Institute (RPMI) medium supplemented with 10 µg/mL macrophage colony-stimulating factor (MCSF) (Genscript) in addition to 10% fetal bovine serum 100 U/mL penicillin, 100 mg/mL streptomycin, 10 mM HEPES, 1 mM sodium pyruvate, 0.1 mM non-essential amino acids, and 2 mM L-glutamine. Differentiation into M2 macrophages was conducted was achieved by the further addition of 10 µg/mL IL-4 (Peprotech Inc.) preceding infection by 24 hours.

### Mice

Pathogen-free C57BL/6 mice, originally obtained from Jackson Laboratories, were bred and maintained in the animal care facility at the University of Kansas. All procedures were approved by the University of Kansas Institutional Animal Care and Use Committee (IACUC) and conducted in accordance with the *Guidelines for the Care and Use of Laboratory Animals*.

### Generation of recombinant JHMV and A59 constructs

Recombinant pBAC-JHMV D1329A constructs were generated using Red recombination. A double base pair mutations in the nsp3 Mac1 were engineered using the Kan^r^-I-SceI marker cassette, as previously described, with primers listed in Table 1. Purified BAC DNA from Cml^r^ Kan^s^ colonies was analyzed by restriction enzyme digest, PCR, and full plasmid sequencing for validation of correct clones. Primers used for validation of correct clones are also listed in Table 1. The recombinant MHV-A59 D1330A mutant was generated, as described previously (55, 56). The D1330A mutation was introduced using site-directed mutagenesis (Q5 Site-Directed Mutagenesis Kit, E0554S, NEB England Biolabs) and sequencing-validated. Full-length viral cDNA was assembled using T4 DNA ligase (M0202L, NEB England Biolabs). Full-length viral RNA was synthesized using an *in vitro* transcription kit (mMESSAGE mMACHINE T7 Transcription Kit, AM1344, Thermo Fisher Scientific)

**TABLE 1:** Linear recombination primers for engineering JHMV-D1329A^*a*^

Primer	Primer sequence (5′−3′)
Forward	TTCATGTATAACACCAAATGTTTGTTTTGTTAAAGGAGCGGTTATAAAGGTTTTGCGC**AGAGGATGACGACGATAAGTAGGG**
Reverse	CGATGACTTCAGCACCAACTCTGCGCAAAACCTTTATAACCGCTCCTTTAACAAAACAAA**GCCAGTGTTACAACCAATTAACC**

^*a*^ Mutated nucleotides are underlined.

### Reconstitution of recombinant pBAC-JHMV-derived virus

Approximately 1 × 10^6^ BHK-MVR cells were transfected with approximately 0.5–1 μg of pBAC-JHMV DNA and 1 µg of pcDNA-MHV-N plasmid or 1 µg of full-length A549 RNA using Polyjet (SignaGen) as a transfection reagent. Virus stocks were created by infecting ~1.5 × 10^7^ 17 Cl-1 cells at an MOI of 0.1 plaque-forming units (PFU)/cell and collecting both cells and supernatant at 16–20 hpi. The cells were freeze-thawed, and debris was removed prior to collecting virus stocks. Virus stocks were quantified by plaque assay on HeLa-MVR cells and sequenced by collecting infected 17 Cl-1 or L929 cells using TRIzol Reagent (Invitrogen). The RNA from JHMV virions was likewise isolated from infected cells and submitted for full genome sequencing (SeqCenter).

### Virus replication assay

Cells were infected at the specified multiplicities of infection (MOIs) via a one-hour adsorption phase with a minimum working volume of DMEM containing virus particles, incubated at 37°C and periodically rocked to ensure complete coverage. Infected cells and supernatants were collected by freeze-thaw at −80°C. Viral titers were determined via plaque assay using HeLa-MVR cells.

### Determination of genome copy number

Supernatants from JHMV-infected 17CL1 cells were freeze-thawed before virus particles were concentrated 100-fold via addition of a 30% sucrose cushion and ultracentrifugation at 27,000 rpm. Viral titers were obtained by plaque assay on HeLa-MVR cells. Viral RNA was harvested from concentrated stocks using TRIzol Reagent (Invitrogen). First-strand cDNA synthesis was performed using the M-MuLV reverse transcriptase (NEB). Quantitative RT-PCR of viral genomic RNA (gRNA) was performed using the Quantstudio 3 Real-Time PCR System (Thermo Fisher), the SYBR Green Universal Master Mix (Thermo Fisher), and the primers listed in Table 2. Determination of copy number was performed by comparing the gRNA levels to standard curves generated from 10-fold dilutions of JHMV-BAC DNA.

**TABLE 2:** qPCR and sequencing primers

Primer and purpose	Primer sequence (5′−3′)
qPCR primers	
*M. musculus* HPRT	
Forward	GCGTCGTGATTAGCGATGATG
Reverse	CTCGAGCAAGTCTTTCAGTCC
MHV gRNA	
Forward	AGGGAGTTTGACCTTGTTCAG
Reverse	ATAATGCACCTGTCATCCTCG
MHV sgRNA	
Forward	TATAAGAGTGATTGGCGTCC
MHV sgRNA6	
Reverse	GTGGGGCCACATTAACCACAAG
MHV sgRNA4	
Reverse	CTAATGCTAGGCGCAGAA
Sequencing primers	
MHV Mac1	
Forward	GGCTGTTGTGGATGGCAAGCA
Reverse	GCTTTGGTACCAGCAACGGAG
MERS Mac1	
Forward	CCGTCTGCACCTCAGACTATC
Reverse	GCAGAGTTGTCTGTGCACAC

### Plaque size measurements

JHMV was serially diluted and plated onto monolayers of indicated cell types in 24-well tissue culture plates. At 18 hpi (DBT) or 20 hpi (L929), cells were imaged using an Accu-Scope EXI-310 equipped with an attached Excelis HDS Digital Imaging System. Plaque diameters were measured using CaptaVision software calibrated with a stage micrometer.

### Plaque reduction assay

Stocks of JHMV *rep*D1329, D1329A, and N1347A were serially diluted and plated in triplicate on cell monolayers of HeLa, DBT, or L929 cells in 24-well tissue culture plates. Plaque formation was assayed at 20 hpi.

### Viral entry assay

In a 24-well plate, DBT, L929, and HeLa cells were seeded at 2.25e5 cells/well and infected with a 100 µL volume of MHV-JHM at an MOI of 1, using a one-hour adsorption phase with regular shaking at 4°C. After the adsorption phase, cells were rinsed twice with PBS before fresh media was added to each well, and plates were subsequently returned to the incubator at 37°C. At 2 hpi, cells were removed from the incubator, rinsed with PBS, trypsinized, and the resulting cell suspension was transferred to 1.5 mL microcentrifuge tubes. Tubes were centrifuged at 7,500 rpm for 5 minutes, and the resulting cell pellets were resuspended in fresh PBS. Cells were rinsed in this fashion three times before a final resuspension in TRIzol Reagent (Invitrogen) frozen at −80°C. RNA extraction was carried out following the manufacturer’s recommended protocol.

### RT-qPCR analysis

Cell monolayers were treated with ice-cold TRIzol Reagent (Invitrogen), and RNA was extracted according to the manufacturer’s recommended protocol. Synthesis of cDNA was conducted using random hexamers (Roche) with M-MuLV reverse transcriptase (NEB) via the manufacturer’s recommended instructions. Quantitation of cDNA transcripts was conducted using the Quantstudio 3 Real-Time PCR System (Thermo Fisher) using the SYBR Green Universal Master Mix (Thermo Fisher) and primers listed in Table 2.

### Mouse infections

For MHV mouse infections, 5- to 8-week-old male and female mice were anesthetized with isoflurane and inoculated intranasally with 1 × 10^4^ PFU recombinant JHMV or intraperitoneally with 500 PFU of A59. To obtain tissue for JHMV titers, mice were euthanized six dpi, brains were removed and homogenized in DMEM. To obtain tissue for A59 virus titers, mice were euthanized on different days post-challenge, and livers were removed and homogenized in DMEM. Viral loads were determined by plaque assay on HeLa-MVR cells.

### Immunoblotting

Total cell extracts were lysed in SDS-based lysis buffer containing Pierce Protease Inhibitor Cocktail (Thermo Scientific), PhosStop phosphatase inhibitor (Roche), 2% β-mercaptoethanol, and 1 mM PMSF. Proteins were resolved on a 10% SDS-PAGE gel, transferred to a polyvinylidene difluoride (PVDF) membrane, incubated with a primary antibody, probed with an infrared (IR) dye-conjugated secondary antibody, visualized using a Li-COR Odyssey Imager (Li-COR), and analyzed using Image Studio software. Primary antibodies used for immunoblotting included anti-MHV N monoclonal antibody (1:10,000), anti-MHV S monoclonal antibody (1:5,000) (29), and anti-actin monoclonal antibody (1:10,000) (clone AC15; Abcam, Inc.). Secondary IR antibodies were purchased from Li-COR.

### Immunofluorescence staining

Cells were seeded at approximately 1 × 10^5^ cells/ well in 8-well chamber, removable slides (ibidi) that were pre-coated with 50 µg/mL collagen for 30 minutes. Cells were infected at an MOI of 0.5. At each time point, cells were rinsed with Hank’s Balanced Salt Solution with 0.01% sucrose (HBSS/Su) and fixed with the addition of 2% paraformaldehyde in HBSS/Su. Permeabilization was performed by rinsing 3× with HBSS/Su containing 0.1% saponin (HBSS/Su/Sap), followed by blocking with 3% normal goat serum in HBSS/Su/Sap. Primary antibodies (mouse α-dsRNA clone rJ2, Millipore) (rabbit α-nsp3, in-house), each diluted 1:1,000 in HBSS/Su/Sap, were added at room temperature for three hours followed by rinsing 3× with HBSS/Su/Sap. Goat-derived secondary antibodies (AlexaFluor 488/555) diluted 1:200 in HBSS/Su/Sap were incubated one hour at room temperature protected from light, followed by rinsing twice with HBSS/Su/Sap, once with HBSS/Su, and once with HBSS. Nuclear staining was achieved via the addition of 300 nM DAPI in HBSS for 30 minutes followed by rinsing twice with HBSS. Slides were finalized by adding Vectashield HardSet Mounting Medium (VectorLabs).

### Confocal microscopy

Images were collected with a Leica DM6-SPE upright laser-scanning confocal microscope using a 63× oil-immersion objective lens with Type F oil. DAPI fluorescence was achieved with a 405 excitation and 430/80 emission. Goat anti-mouse ALEXA 488 was imaged with 488 excitation 500/40 emission. Goat anti-rabbit ALEXA 555 was imaged with 561 excitation and 570/90 emission. Images were acquired using Leica LasX software.

### Quantification of confocal immunofluorescence

Quantification of protein staining was automated using a custom macro written in FIJI (ImageJ v.1.54f) (57). For each image, a median 3D filter was applied to multi-channel Z-stacks (x = 1, y = 1, z = 1 for DBT; x = 2, y = 2, z = 2 for BMDM) after which maximum intensity Z-projections were generated, and the channels were split. Regions of interest (ROIs) were generated from these single-channel duplicates by automated thresholding via the Moments, Triangle, and Renyientropy methods for the DAPI, dsRNA/N protein, and nsp3 channels, respectively. Resulting binary masks were applied to the original Z-projections, and fluorescence intensity of staining was measured within regions of interest (ROIs) on the relevant channel. The total area of each ROI was also recorded, and the integrated density (IntDen) displayed is a product of the mean fluorescence intensity and the ROI area.

### Protein expression and purification

Recombinant proteins were produced, as previously described (35).

### *In vitro* de-MARylation assay

MARylated PARP10 was generated by incubating purified GST-PARP10 with β-nicotinamide adenine dinucleotide (β NAD^+^) (Millipore-Sigma) in a reaction buffer (50 mM HEPES, 150 mM NaCl, 0.2 mM DTT, and 0.02% NP-40). MARylated PARP10 was aliquoted and stored at −80°C. Purified SARS-CoV-2 Mac1 WT and D-A were co-incubated with MARylated PARP10 for the indicated times at 37°C at a ratio of 1 µM Mac1 to 5 µM PARP10 in reaction buffer (50 mM HEPES, 150 mM NaCl, 0.2 mM DTT, and 0.02% NP-40). Reactions were halted by adding 2× Laemmli sample buffer containing 10% β-mercaptoethanol. Samples were then heated for five minutes at 95°C before loading into a Bolt 4%-12% Bis-Tris Plus Gel in MES running buffer (Thermo Fisher). Direct protein detection was conducted via staining on a separate, identical gel using ReadyBlue Protein gel stain (Sigma). Transfer to polyvinylidene difluoride (PVDF) membrane was achieved via the iBlot 2 Dry Blotting System (Thermo Fisher). I mmunoblotting of the MAR and GST signal was conducted using anti-mono ADP-ribose binding reagent MABE1076 (Sigma) and an anti-GST monoclonal antibody MA4-004 (Thermo Fisher). Detection of primary antibodies was conducted using secondary infrared anti-rabbit and anti-mouse antibodies (LI-COR Biosciences). Samples were visualized using the Odyssey M Imaging System (LI-COR Biosciences).

### Isothermal titration calorimetry

All ITC titrations were performed on a MicroCal PEAQ-ITC instrument (Malvern Pananalytical Inc., MA). All reactions were performed in 20 mM Tris (pH 7.5) and 150 mM NaCl using 100 µM of each macrodomain protein at 25°C. Titration of 2 mM ADP-ribose or ATP (MilliporeSigma) contained in the stirring syringe consisted of a single 0.4 µL injection, followed by 18 consecutive injections of 2 µL. Thermogram data were analyzed using one set of binding sites model in MicroCal ITC software to obtain all fitting model parameters for the experiments. MERS-CoV and SARS-CoV-2 WT protein ITC data were previously published (35). These experiments were performed alongside the mutant proteins, thus serving as appropriate controls.

### Differential scanning fluorimetry

A thermal shift assay using differential scanning fluorimetry (DSF) was performed with LightCycler 480 Instrument (Roche Diagnostics). In total, a 15 µL mixture containing 8× SYPRO Orange (Invitrogen) and 10 µM macrodomain protein in buffer (20 mM Hepes, NaOH, pH 7.5) with varying concentrations of ADP-ribose were mixed on ice in 384-well PCR plate (Roche). Fluorescence signals were measured from 25 to 95°C in 0.2°C/30 s steps (excitation, 470–505 nm; detection, 540–700 nm). Data evaluation and Tm determination were performed using the Roche LightCycler 480 Protein Melting Analysis software, and data fitting calculations were conducted using single-site binding curve analysis on GraphPad Prism.

### AlphaScreen assay

The AlphaScreen reactions were carried out in 384-well plates (Alphaplate, PerkinElmer, Waltham, MA) in a total volume of 40 µL in buffer containing 25 mM HEPES (pH 7.4), 100 mM NaCl, 0.5 mM TCEP, 0.1% BSA, and 0.05% CHAPS. All reagents were prepared as 4× stocks, and 10 µL volume of each reagent was added to a final volume of 40 µL. After one-hour incubation at RT, streptavidin-coated donor beads (7.5 µg/mL) and nickel chelate acceptor beads (7.5 µg/mL) (PerkinElmer AlphaScreen Histidine Detection Kit) were added under low light conditions, and plates were shaken at 400 rpm for 60 minutes at RT protected from light. Plates were kept covered and protected from light at all steps and read on BioTek plate reader using an AlphaScreen 680 excitation/570 emission filter set. For data analysis, the percent inhibition was normalized to positive (DMSO+labeled peptide) and negative (DMSO+macrodomain + peptide, no ADPr) controls.

### Statistical analysis

A Student’s *t*-test was used to analyze differences in the mean values between two groups; for multiple group comparisons, an ordinary one-way ANOVA was used. All results are expressed as means ± standard errors of the means (SEM) unless stated as standard deviation (SD) or as geometric means ± 95% confidence interval. *P* values of ≤ 0.05 were considered statistically significant (*P ≤ 0.05; **P ≤ 0.01; ***P ≤ 0.001; ****P ≤ 0.0001; ns, not significant).

## Data Availability

All data associated with this manuscript is available through the data repository FigShare at 10.6084 /m9.figshare.c.7755170.

## SUPPLEMENTAL MATERIAL

Supplemental figures jvi.00623-25-s0001.pdf Fig. S1 to S3.

ASM does not own the copyrights to Supplemental Material that may be linked to, or accessed through, an article. The authors have granted ASM a non-exclusive, world-wide license to publish the Supplemental Material files. Please contact the corresponding author directly for reuse.
